# Is Inducible Nitric Oxide Synthase (iNOS) Promising as a New Target Against Pulmonary Hypertension?

**DOI:** 10.3390/antiox14040377

**Published:** 2025-03-21

**Authors:** Piotr Ryszkiewicz, Eberhard Schlicker, Barbara Malinowska

**Affiliations:** 1 Department of Experimental Physiology and Pathophysiology, Medical University of Bialystok, Mickiewicz Str. 2A, 15-222 Bialystok, Poland; 2 Department of Pharmacology and Toxicology, University of Bonn, Venusberg Campus 1, 53127 Bonn, Germany; e.schlicker@uni-bonn.de

**Keywords:** nitric oxide, inducible nitric oxide synthase, nitrosative stress, pulmonary hypertension, cardiovascular system, pulmonary arterial hypertension, enzyme inhibition, oxidative stress, inflammation, animal models

## Abstract

Pulmonary hypertension (PH) is a progressive disease characterized by elevated blood pressure in the pulmonary arteries, associated also with inflammation and oxidative stress. Inducible nitric oxide synthase (iNOS) is one of the key mediators of inflammation and immune system activation. Although preclinical studies mostly suggest a detrimental role of iNOS overactivation in PH, there is a lack of exhaustive analyses and summaries. Therefore, this literature overview aims to fill this gap. The involvement of iNOS in the pathogenesis of the four main clinical groups of PH is discussed to assess whether targeting iNOS could be a promising way to treat PH. iNOS expression patterns in the organs primarily affected by PH are analyzed both in animals and in humans. Consequently, the effectiveness of pharmacological iNOS inhibition and/or iNOS gene deletion is discussed and compared, also with reference to the activity of constitutive NOS isoforms, particularly endothelial NOS (eNOS). Overall, our overview suggests that selective iNOS inhibitors could be considered as a novel treatment strategy for PH, as decreases in right ventricular and pulmonary artery pressure, the alleviation of ventricular hypertrophy, and improvements of pulmonary and cardiac function were observed, among others. Nevertheless, further research efforts in this area are needed.

## 1. Introduction

Pulmonary hypertension (PH) comprises a group of disorders characterized by a mean pulmonary artery pressure (mPAP) over 20 mmHg, measured at rest via right heart catheterization [1,2]. The classification of PH encompasses pulmonary arterial hypertension (PAH) and PH associated with left heart disease, lung diseases, hypoxia or pulmonary artery obstructions (Table 1) [1]. In PH pathogenesis, many processes are involved, which are so complex that differences exist not only between individual clinical groups, but also within them [3]. However, a few linking features shall be highlighted, i.e., elevated blood pressure in the pulmonary arteries, leading to increased pulmonary vascular resistance (PVR), vascular remodeling, right ventricular (RV) dysfunction, excessive oxidative stress, and inflammation [1,2,3,4,5,6].

**Figure 1:**
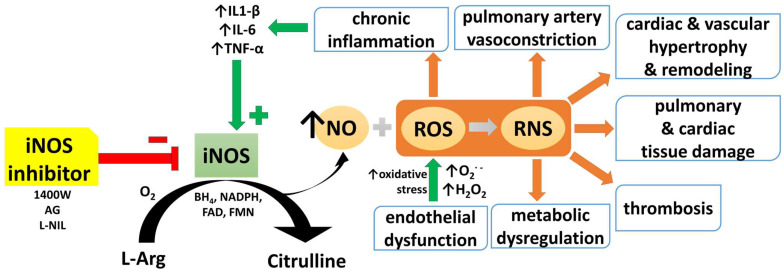
Contribution of **inducible nitric oxide synthase** (**iNOS**) overexpression to the pathological processes involved in **pulmonary hypertension** (**PH**). This schematic diagram explains the basis of the enzymatic activity of iNOS and gives a general perspective on the possibly detrimental role of iNOS-derived nitric oxide (NO) in the progression of PH. Excessive amounts of NO, generated in response to iNOS induction by proinflammatory cytokines (IL-1β, IL-6, TNF-α), are prone to interact with reactive oxygen species (ROS), giving peroxynitrite and other reactive nitrogen species (RNS, orange rectangle), which directly or indirectly (e.g., via decreased NO bioavailability) promote the mechanisms underlying the development of PH (blue rectangles). Abbreviations: 1400W, N-(3-(aminoethyl)benzyl)acetamidine; AG, aminoguanidine; BH_4_, tetrahydrobiopterin; FAD, flavin adenine dinucleotide; FMN, flavin mono¬nucleotide; iNOS, inducible nitric oxide synthase; IL-1β, -6, interleukin 1β, -6; L-Arg, L-arginine; L-NIL, L-N6-(1-iminoethyl)-lysine; NADPH, reduced nicotinamide adenine dinucleotide phosphate; O_2_^•−^, superoxide anion; TNF-α, tumor necrosis factor alpha; +, stimulation; –, inhibition; ↑, increase.

At the crossroads of the latter two processes stands inducible nitric oxide synthase (iNOS, NOS2), a key mediator of immune activation. Nitric oxide (NO), the product of its catalytic activity, serves primarily as a potent vasodilator, and plays a critical role in maintaining vascular homeostasis and regulating pulmonary vascular tone [7]. However, its excessive production seems to be a double-edged sword, as NO’s reaction with superoxide radicals leads to the formation of peroxynitrite, a highly reactive species that promotes oxidative stress and endothelial dysfunction (Figure 1) [8].

In this review, the role of NO in cardiopulmonary diseases has been highlighted (Section 2), and characteristics of the main clinical groups of PH have been described (Section 3). To get an idea of whether iNOS might serve as a potential therapeutic target in PH, we have searched the literature for changes in its expression in animal models (Section 5). In particular, we compared the efficacy of iNOS inhibitors (Section 6) and of genetic iNOS ablation (Section 7). In the final part, the (so far limited) data from clinical studies are presented (Section 8).

## 2. Nitric Oxide and Its Role in Cardiopulmonary System

### 2.1. Nitric Oxide

Nitric oxide is a free gaseous radical with an unpaired electron. NO is a key mediator in the cardiopulmonary system and serves as a paracrine and autocrine signaling molecule, which participates in vasodilation, smooth muscle relaxation, neurotransmission, and immune responses [7,8,9,10,11]. It also possesses anti-inflammatory, anti-thrombotic, and anti-proliferative properties, which contribute to the overall maintenance of cardiovascular and pulmonary health state [10,11]. NO is produced by the oxidation of amino acid L-arginine (L-Arg) to L-citrulline (Figure 1). This reaction is catalyzed by each of the three isoforms of nitric oxide synthase (NOS): neuronal nitric oxide synthase (nNOS, NOS1), endothelial nitric oxide synthase (eNOS, NOS3) (both constitutively expressed), and iNOS, which is expressed only when activated by certain stimuli [7,11]. NO can also be alternatively formed from the nitrite anion (NO_2_^–^) through a NOS-independent mechanism [12]. Despite its short half-life, NO is critical for the proper functioning of cells and for maintaining vascular homeostasis [10,13].

The biological role of NO is multifaceted and depends on, e.g., its concentration, the activity of certain enzymes (e.g., arginase, which converts the NOS substrate, L-Arg, to ornithine and urea, thereby affecting NOS activity), and the presence of reactive oxygen species [8]. However, the border between beneficial and detrimental aspects of NO is very thin, and to date not entirely clear. Although NO, at lower concentrations, plays a positive role in regulating various biological processes, it appears to be detrimental at higher levels. For instance, NO interaction with superoxide (at a diffusion-limited rate) gives a potent oxidative and nitrative agent, peroxynitrite (ONOO^−^), a fundamental mediator of tissue injury [14]. Unless neutralized to a harmless nitrate, peroxynitrite can exert serious damage to DNA, alter DNA repair processes, irreversibly inactivate important cellular proteins (e.g., via nitration of tyrosine), or initiate the production of other cytotoxic molecules [8,10]. NO participates in posttranslational modifications of proteins, e.g., S-nitrosylation of cysteine, nitration of tyrosine, and nitrosylation of prosthetic groups [10]. Excessive amounts of NO are also prone to interact with transition metals, e.g., iron in heme and cobalt in cobalamine, thereby disrupting the biological activity of metal-containing complexes [7,11]. However, the latter properties, if considering them as a part of the immune system response against pathogens or tumor cells, are found to be beneficial [7,8,15].

Soluble guanylate cyclase (sGC) is the physiological executor of NO functions. Activated by NO, sGC increases the intracellular levels of cyclic guanosine-3′,5′-monophosphate (cGMP), which results in the activation of various intracellular effector molecules, e.g., the activation of cGMP-gated calcium-sensitive potassium channels, leading to the inhibition of the sarcoplasmic reticulum-mediated release of calcium. A subsequent decrease in intracellular calcium inhibits the phosphorylation of myosin and, eventually, vasoconstriction [10,16].

### 2.2. The Role of NOS in the Cardiopulmonary System

All three isoforms of NOS were identified in the human respiratory system. They concomitantly participate in the regulation of the above-mentioned physiological processes via complementary NO synthesis. eNOS is localized in the cellular membrane of endothelial cells of pulmonary blood vessels, but also in epithelial cells of trachea, bronchi, and alveoli [8,17]. nNOS is constitutively expressed in epithelium, and in the inducible non-adrenergic–non-cholinergic autonomic system (iNANC), where it participates in the regulation of airway smooth muscle tone as an inhibitory neurotransmitter [8,18]. iNOS, similarly to nNOS, is a cytoplasmic enzyme. It generates higher quantities of NO than constitutively express isoforms (micromolar vs. nanomolar levels, respectively) in a continuous release manner [7,8]. iNOS is expressed while stimulated by proinflammatory stimuli in various types of cells, e.g., smooth muscle cells, cardiac myocytes, hepatocytes, chondrocytes, glial cells, astrocytes, neurons, and microglia, as a type of defense mechanism [7]. Within the pulmonary system, it is expressed under such conditions in alveolar macrophages, the epithelium of the proximal and terminal bronchioles, alveolar epithelial type II cells, lung fibroblasts, bronchial and vascular smooth muscle cells, mast cells, neutrophils, and the endothelium [8]. iNOS is considered a key mediator of immune activation and inflammation. Proinflammatory cytokines (i.e., interleukin-1β (IL-1β), interferon-γ (IFN-γ), tumor necrosis factor-α (TNF-α), Figure 1) and bacterial lipopolysaccharide (LPS) are the main endogenous and exogenous inducers of iNOS, respectively. The combination of these stimulators might generate a synergistic effect [7].

### 2.3. iNOS—Protective or Harmful?

The dysregulation or overexpression of iNOS is typical for many pathological states, e.g., cardiovascular diseases, sepsis (and septic shock), various types of pain, diabetes mellitus, and neurodegenerative disorders [13,19,20,21,22]. Cancer is a disease sustained by the high expression of iNOS [20,21]. High iNOS expression has also been determined in patients infected with Mycobacterium tuberculosis, Plasmodium falciparum, and HIV [7]. Also, insulin-sensitive tissues induce iNOS, which might be related to insulin resistance development and diabetes mellitus [11,23,24]. Excessive NO production is also observed in many complex diseases associated with inflammation, e.g., Alzheimer’s disease, Parkinson’s disease, multiple sclerosis, rheumatoid arthritis, inflammatory bowel disease, and celiac disease [10].

In the case of cardiopulmonary diseases, a multifaceted role of iNOS is suggested [25,26]. For instance, iNOS plays a pivotal role in the recruitment and activation of macrophages during the inflammatory phase of acute lung injury [27]. It contributes to heart failure with a preserved ejection fraction [28]. The inhibition of iNOS activity alleviated chronic allergic pulmonary inflammation in guinea pigs [29] and lung remodeling in mice [30]. However, in myocardial ischemia–reperfusion injury, both detrimental and beneficial effects of iNOS have been described. On the one hand, NO derived from upregulated iNOS might attenuate cardiac contractile function and increase oxidative stress and myocardial apoptosis. On the other hand, during ischemic preconditioning, hypoxia inducible factor 1α (HIF-1α) signaling enhances iNOS-derived NO and increases levels of TNF-α and cyclooxygenase-2 (COX-2)-dependent prostanoids, which lead to myocardial protection. The elimination of oxidative stress might contribute to switching iNOS from harmful to protective [31]. Additionally, a dual role of iNOS has already been established in postischemic cardiac remodeling (protective in the context of preconditioning and deleterious during chronic exposure to proinflammatory stimuli) [32].

## 3. Pulmonary Hypertension (PH)—Short Characteristics of the Main Clinical Groups

### 3.1. Pulmonary Arterial Hypertension

Pulmonary arterial hypertension (PAH, group 1 PH, Table 1), a subtype of PH that primarily affects the pulmonary vasculature, is a progressive and devastating chronic disease. Long-standing pressure overload in the pulmonary artery leads to RV hypertrophy, RV failure, and premature death, especially if this condition remains untreated [33,34,35]. PH should not be mixed up with PAH, as these terms are not exactly synonymous. Throughout this review, according to Tabima et al. (2012) [12], we will refer to pulmonary arterial hypertension as PAH in the context of human group 1 disease and to pulmonary hypertension as PH in the context of group 2–5 disease in patients, and in the context of all preclinical animal models.

The pathogenesis of PAH involves multiple processes, interconnected at the molecular, cellular, and tissue levels, such as endothelial dysfunction [36,37], excessive vasoconstriction [38,39,40,41], vascular remodeling [3,42,43], oxidative stress, inflammation and immune dysregulation [40,44,45], metabolic dysregulation [46,47], and thrombosis [39,48], i.e., processes driven by iNOS overexpression (Figure 1). While most cases of PAH are sporadic, a small proportion of individuals inherits the disease through genetic factors. Mutations in bone morphogenetic protein receptor 2 gene (*BMPR2*), a member of the transforming growth factor β (TGF-β) family, dramatically increase the risk of developing heritable PAH, and lead to dysregulations in signaling pathways involved in cell growth, differentiation, and survival [35,37,40,49,50].

Currently, Food and Drug Administration- (FDA) or European Medicines Agency (EMA)-approved drugs for PAH therapy mainly comprise vasodilators, including (1) stimulators of sGC (riociguat) or inhibitors of phosphodiesterase 5 (sildenafil, tadalafil) [51], which target the NO/cGMP pathway, (2) synthetic prostacyclin analogues (iloprost, treprostinil) or prostacyclin IP receptor agonists (selexipag) and (3) endothelin-A receptor antagonists (ambrisentan, bosentan, macitentan). Additionally, amlodipine, a calcium channel blocker, might be considered as a treatment option in patients with positive vasoreactivity test results [1,34,52,53,54]. It is important to note that not all PAH patients require or respond to the same therapies, thus individualized assessment, taking into account the severity of the disease and patient-specific factors, appears the optimum way to achieve better outcomes [1,5]. PAH is still considered an incurable disease with a high mortality rate (5-year survival rate ~50%; in end-stage disease, lung transplantation remains the only treatment option), and drugs currently available on the market have not been shown to reduce mortality in randomized, controlled clinical trials [5]. So, there is an urgent need to define new targets and investigate other drug candidates [5,34,35,55].

### 3.2. Pulmonary Hypertension Due to Left Heart Disease

Pulmonary hypertension due to left heart disease (PH-LHD, group 2 PH, Table 1) is characterized by elevated blood pressure in the pulmonary circulation, which results from dysfunction or pathology of the left side of the heart. This condition typically arises as a consequence of left ventricular (LV) systolic or diastolic dysfunction, valvular heart diseases, or myocardial diseases [1]. The underlying mechanism involves increased left atrial pressure, which is transmitted backward into the pulmonary vasculature, leading to an elevation in PAP, and pulmonary vascular remodeling [56,57,58,59]. This imposes an additional burden on the RV, ultimately contributing to a progressive and often debilitating clinical course [60]. The pathophysiology of PH-LHD remains insufficiently comprehended. Pulmonary vascular remodeling may stem from (1) heightened wall stress attributable to elevated left atrial pressure; (2) reduced shear stress in the pulmonary vascular bed induced by hemodynamic congestion; (3) endothelial dysfunction prompted by comorbidities, leading to direct harm to the pulmonary microvasculature, and/or (4) the influence of risk factors for PAH [61].

Although the incidence of PH-LHD is the highest of all clinical PH groups (65–80% of cases [1]), there is no specific treatment for this condition, and advantages of treatment schedules approved for PAH could not be shown in randomized clinical trials [58,61]. The management of PH-LHD as of now encompasses targeting the underlying left heart pathology to alleviate left heart dysfunction, reduce PVR, and improve overall cardiopulmonary function [1]. Treatment protocols may include diuretics, angiotensin-converting enzyme inhibitors, beta-blockers, and, in select cases, surgical interventions, e.g., valve replacement [1,62,63].

### 3.3. Pulmonary Hypertension Associated with Lung Diseases and/or Hypoxia

Pulmonary hypertension is a relatively common complication of chronic lung diseases (group 3 PH, Table 1), such as chronic obstructive pulmonary disease (COPD) or idiopathic pulmonary fibrosis (IPF). It affects ~40% of patients suffering from each of these diseases [64,65]. Fibrosis is also considered to be involved in PAH pathogenesis (see above). Nonetheless, the use of PAH-approved drugs in patients with group 3 PH is very limited, and the evidence for clinical benefits of such a treatment remains unclear and conflicting [1].

COPD is a progressive respiratory condition characterized by the limitation of airflow, and is primarily caused by environmental factors, such as exposure to noxious particles or gases, most commonly cigarette smoke, with the concomitant influence of several genetic factors [66,67]. The pathogenesis of COPD involves inflammation, increased oxido-nitrosative stress, structural changes in the airways due to imbalances between proteolytic activity and anti-proteolytic defense, uncontrolled autophagy, and/or enhanced apoptosis [66,67,68,69]. IPF is a condition in which the lungs become scarred over time. Although the cause is unknown (leading to the term “idiopathic”), the pathogenesis may comprise environmental and/or endogenous injury to alveolar epithelium, resulting in the promotion of extracellular matrix deposition, increased cell death, and/or the dysregulation of epithelial–fibroblast cross-talk [70,71,72]. Chronic hypoxia is a prolonged deficiency of oxygen in the tissues, often stemming from conditions such as COPD and interstitial lung disease, but also as a result of high-altitude (≥2500 m) living. Hypoxic pulmonary vasoconstriction (HPV) is a physiological homeostatic mechanism that promotes the constriction of pulmonary vessels in response to low oxygen levels [73]. However, its dysregulation or excessive activation may contribute to severe cardiopulmonary complications, e.g., high-altitude PH and high-altitude pulmonary edema [73,74].

### 3.4. Pulmonary Hypertension Associated with Pulmonary Artery Obstructions

Chronic thromboembolic pulmonary hypertension (CTEPH; group 4 PH; Table 1) is a form of PH characterized by the persistent obstruction of pulmonary arteries due to remodeling following thromboembolic events. In acute pulmonary embolism (APE), blood clots that typically originate in the deep veins of the legs or pelvis migrate to the pulmonary arteries, causing vascular obstruction. In ~4% of the patients with APE, the thromboembolic material fails to resolve [75], leading to chronic pulmonary vascular changes [76]. The pathogenesis involves the formation of fibrotic material in the pulmonary arteries, leading to increased PVR and elevated (m)PAP. This results in right heart strain and, if left untreated, may progress to right heart failure [76]. The gold standard multi-modal approach for alleviating pulmonary artery obstruction in individuals with CTEPH comprises pulmonary endarterectomy, balloon pulmonary angioplasty, and pharmacotherapy [1]. The aim is to decrease PVR, alleviate PH, and mitigate RV dysfunction [77].

## 4. Study Selection Criteria

To check the contribution of iNOS activation to PH development, we conducted an extensive search in the PubMed database (closed on 11 March 2025). Firstly, we checked for the clinical evidence of iNOS involvement in PH. The search was performed by combining the phrases regarding respective clinical (sub)groups of PH (Table 1) with “inducible nitric oxide synthase”, “iNOS” or “NOS2”, and “humans” or “patients”. Then, after the appropriate animal models mimicking those four clinical groups had been identified (see Section 5.1), we searched for preclinical studies in which the expression of iNOS in the organs primarily affected by PH was assessed, iNOS inhibitors were administered, or animals with genetic NOS depletion were used. The following example phrases were used during the search: “pulmonary hypertension monocrotaline iNOS (or NOS2)”, “pulmonary hypertension hypoxia iNOS (or NOS2)”, “pulmonary hypertension emphysema iNOS (or NOS2)” etc. Ultimately, 112 publications were selected for this review, as summarized in Section 5.2, Section 6, Section 7 and Section 8, respectively. Additional references were included to provide more background on NOS and NO signaling in the cardiopulmonary system, human PH, and respective animal models of this disease.

## 5. iNOS Expression Patterns in Preclinical Models of PH

### 5.1. A Brief Overlook on Animal Models

Due to the complexity of P(A)H pathogenesis, briefly noted above, animal models are useful tools for investigating the complicated molecular pathways and developing novel therapeutic interventions. In this section, only a brief description of the preclinical PH models considered in this review will be given. For more details, the reader is referred to some excellent reviews [78,79,80,81,82,83,84].

One of the most frequently used models is chronic hypoxia-induced PH (Hx-PH) [79]. However, a prolonged deficiency of oxygen is also associated with high-altitude living or chronic lung diseases, such as COPD. Thus, it appears quite challenging to accurately classify whether the Hx-PH model under study mimics human PAH (clinical group 1) rather than high-altitude PH (clinical group 3). For the purpose of this review, we assume that the hypoxic model predominantly mimics PAH. The combination of chronic hypoxia with the administration of the angiogenesis inhibitor Sugen (SU5416), a vascular endothelial growth factor receptor (VEGFR) antagonist, represents another model, in which concentric laminar and plexiform lesions (i.e., glomus-like structures, the walls of which consist of fibrous tissue covered by endothelial cells) typical of human PAH are developing [79].

The monocrotaline (MCT) model is also commonly used to induce PH in rodents, but, importantly, it does not entirely mimic the complexity of human PAH pathobiology as, e.g., plexiform lesions are not detected [79]. Furthermore, the gradual and chronic nature of human PAH is not reflected well, as MCT-PH often develops very rapidly [85,86], and the variability of the individual response to this alkaloid is quite high [79,85,86]. Moreover, the toxicity of MCT is not limited to the pulmonary vasculature only, but also affects other organs and tissues, e.g., liver and kidneys; so, non-specific effects may be elicited that confound the interpretation of the experimental results [79].

Several models of PH-LHD (group 2 PH) have been proposed to date [80,81]; they induce increased pressure in the left heart artificially, e.g., via transverse aortic constriction (TAC). This, in turn, triggers a heightened pressure response in the pulmonary artery through backward transduction. Moreover, obesity-induced models are applied, as among the majority of patients with PH-LHD, the combination of metabolic syndrome, hypertension, hyperlipidemia, and/or diabetes mellitus is very common [1,87]. Both models mimic certain aspects of left heart failure with preserved ejection fraction (HFpEF) [80].

With respect to preclinical models resembling clinical group 3 PH, we will refer to COPD induced either by cigarette smoke or elastase instillation. Both models share some notable similarities, i.e., the development of emphysema-like changes, excessive oxidative stress, and inflammation in the respiratory tract [88]. Additionally, one model of IPF will be considered, i.e., pulmonary fibrosis induced by intratracheal instillation of bleomycin. Progressive fibrosis, excessive inflammation and oxidative stress, and a decline in pulmonary function (i.e., reduced lung compliance and impaired gas exchange) are its main features [82,83].

CTEPH (group 4 PH) is mimicked in animal experimental models by permanent ligation of the left PA, the induction of blood clots in vivo, and the administration of autologous thrombi prepared in vitro or exogenous particles of a non-thrombotic nature [84]. In our review, we will refer to the latter method only.

### 5.2. Changes in iNOS Expression in Preclinical Studies

The overall picture of iNOS expression in PH animals is shown in Table 2. One should be aware that we focused on the changes in iNOS expression in organs primarily affected by PH, namely, lungs, heart, and pulmonary vasculature, across a variety of models mimicking the respective clinical groups of PH. We believe that this is a suitable approach to establish a framework for formulating more general conclusions. However, one should also keep in mind that iNOS expression is regulated or influenced by a complex interplay of multiple factors, including the experimental conditions, the pathophysiological and molecular characteristics of the model used, and ultimately, by the interaction with other signaling pathways. As shown in Figure 1, typical P(A)H features can be connected with an increase in iNOS activity/expression (blue rectangles).

The assessment of the localization of iNOS expression varies, depending on the method used, i.e., western blotting, reverse transcription polymerase chain reaction (RT-PCR), quantitative polymerase chain reaction (qPCR), in-situ hybridization or immunohistochemistry. Considering lung tissue and vasculature, iNOS expression was determined either in whole lung homogenates [89,90,91,92,93,94,95,96,97,98] or in the respiratory tract, i.e., trachea, airway epithelia and intraparenchymal airways [99,100,101], alveoli [101,102,103], lung macrophages [103,104], or pulmonary vasculature [102,105,106,107]. Taking into account the heart [108], the expression was assessed mainly in the myocardium [105,109,110], but also in fibroblasts [111], from either the right [112] or the left [113] ventricle.

Unlike in most control tissues, in which iNOS expression was negligible, experimental PH in most cases increased iNOS mRNA or protein expression. Increases in iNOS expression were observed in all experimental models of the different PH groups. The histological distribution of iNOS overexpression is important, as it plays a role in the pathogenesis of the particular forms of PH. In early phases of chronic hypoxic exposure, iNOS induction appeared predominantly in the smooth muscle layer of pulmonary arteries (i.e., media), and, to a lesser extent, in endothelium (i.e., intima) and adventitia [100,101,114,115,116]. During prolonged hypoxia, iNOS expression in the pulmonary vascular wall returned to nearly undetectable baseline levels, but was continuously present in the airway epithelium [100]. Flow/wall shear stress may also be the cause of increased iNOS expression [117,118]. During chronic tobacco smoke exposure, the upregulation of the iNOS protein was more prominent in the pulmonary vasculature than in the alveoli or bronchi [119], and was also observed in systemic blood vessels, i.e., aorta [120]. Pulmonary fibrosis induced by bleomycin was also associated with increased iNOS expression in lungs of rats [121,122] and mice [123,124,125,126]. The administration of embolizing particles, mimicking certain aspects of human group 4 PH, resulted in iNOS induction in lungs [127] and pulmonary vasculature [128].

Interestingly, in a few cases (Table 2), no changes in iNOS expression were observed [129,130,131,132,133,134]. In the study by DeMarco et al. (2009), this lack of effect was explained by the fact that iNOS transcript levels were highly variable and, as a consequence, the detection of significant changes was impaired [130]. Moreover, shifts to the expression of other NOS isoforms were observed [130]. In MCT-treated rodents, iNOS upregulation was accompanied by simultaneous eNOS downregulation [102,135]. On the other hand, prolonged exposure to low oxygen levels enhanced the expression of all three NOS isoforms in pulmonary vessels [136,137,138], but only the increase in iNOS expression was prone to coincide with the onset of hypoxic pulmonary vascular remodeling and PH development [100,139]. By contrast, Camelo et al. (2012) showed the overexpression of iNOS with the concomitant downregulation of eNOS in pulmonary artery endothelial cells, alveolar and interstitial macrophages [131]. The overactivation of iNOS, with the concomitant downregulation of eNOS, was also detected in the pulmonary vasculature of smoke-exposed mice [68,69,119,140,141]. However, when fetuses or newborns were subjected to hypoxia, this type of relationship did not hold true. In newborn PH piglets, both eNOS and iNOS activities in pulmonary artery homogenates were decreased [134]. This might suggest that PH in newborns, in contrast to adults, is associated with decreased NO production [132,134]. Nevertheless, Evans et al. (2012) showed that chronic hypoxic exposure enhances iNOS and decreases eNOS expression in the ventricles of fetal guinea pig hearts [142]. Increased iNOS expression was also found in a model mimicking portal PH [143].

Across the analyzed studies (Table 2), iNOS expression was determined mainly in tissues collected at the end of the experiment, i.e., at the time of full development of PH. However, the proper selection of the time points for the assessment of iNOS expression in the experimental protocol is of high importance, as it might affect the results obtained. For instance, after four-day hypoxia, a striking iNOS expression was observed in the lungs of hypoxic rats, but these levels returned to baseline after 20 days of hypoxia [100]. In the study by Guo et al. (2023b), the expression became significant at 1 week, and reached its peak at 3 weeks after TAC surgery [144]. The induction of iNOS expression in pulmonary vasculature in response to exposure to cigarette smoke was evident already 2 h after the beginning of the exposure, and was still present 24 h after its termination [99]. In an elastase-induced model of pulmonary emphysema, iNOS lung expression gradually increased over time, and was already higher than in the respective control 24 h after the onset of elastase application [103]. Similarly, in hypertrophied RV, iNOS expression increased gradually over time during 8-, 15-, and 21-day hypoxia [110].

**Table 2:** Changes in inducible nitric oxide synthase (iNOS) mRNA and protein expression in preclinical models representing various clinical groups of pulmonary hypertension (PH) in comparison to the respective controls *.

Disease Entity	Model	Species	Tissue	Changes in iNOS mRNA/Protein Expression	References
**Models of disease entities within group 1 PH**					
PAH	MCT-induced PH	rat	lungs	↑	[90,91,92,93,94,136,137]
				↔	[129]
			alveoli	↑	[102]
			PA	↑	[102]
			RV (myocardium)	↑	[109,112,135]
			RV (fibroblasts)	↑	[111]
	Hx-induced PH	rat	lungs	↑	[95,96,97,114,115,139,145]
			lung macrophages	↑	[104]
			alveoli	↑	[101]
			PA (endothelium, smooth muscle cells)	↑	[100,101,104,106,116,146]
			hearts (whole)	↑	[108]
			RV myocardium	↑	[110]
			LV myocardium	↑	[110]
		mouse	lungs	↑	[98]
	Sugen-Hx-induced PH	rat	pulmonary vessels	↑	[105]
			RV myocardium	↑	[105]
	Ren2 rat model	rat	lungs	↔	[130]
portal PH	portal vein ligation	rat	lungs	↑	[143]
PPHN	Hx-induced PH	rat ♀ (pregnant)	lungs	↔	[132]
		pig (newborn)	lungs	↔	[131]
			PA	↔ (membrane fraction)	[134]
				↓ (cytosolic fraction)	
		guinea pig ♀ (pregnant)	LV	↑	[113]
**Models of disease entities within** **group 2 PH**					
HFpEF	TAC	mouse	lungs	↑	[89]
			RV myocardium	↑	[144,147]
	metabolic (obesity)-HF	rat	lungs	↔	[133]
			PA	↑	[107]
**Models of disease entities within group 3 PH**					
COPD	elastase-induced lung emphysema	mouse	lungs (macrophages, alveolar wall, alveolar epithelium)	↑	[103,141]
	cigarette smoke-induced lung injury	rat	trachea, intraparenchymal airways	↑	[99]
			PA	↑	[99]
			aorta	↑	[120]
		mouse	lungs	↑	[119,140,148]
			bronchi	↑	[119,148]
		guinea pig	lungs	↑	[149]
IPF	bleomycin-induced pulmonary fibrosis	rat	lungs	↑	[121,122]
			PA	↑	[93]
		mouse	lungs	↑	[123,124,125,126,150]
**Models of disease entities within group 4 PH**					
chronic thromboembolic PH	administration of embolizing particles	broiler chicken	lungs	↑	[127]
		rabbit	pulmonary vessels, alveoli	↑	[128]

* Experiments were performed on male animals, if not stated otherwise. COPD, chronic obstructive pulmonary disease; HF, heart failure; HFpEF, heart failure with preserved ejection fraction; Hx, chronic hypoxia; iNOS, inducible nitric oxide synthase; IPF, idiopathic pulmonary fibrosis; LV, left ventricle; MCT, monocrotaline; PA, pulmonary artery; PAH, pulmonary arterial hypertension; PH, pulmonary hypertension; PPHN, persistent pulmonary hypertension of the newborn; RV, right ventricle; TAC, transverse aortic constriction; ♀ female; ↓, decrease; ↑, increase; ↔ no change(s).

## 6. Pharmacological iNOS Inhibition—Promising or Discouraging Way to Treat PH?

Considering the changes in iNOS expression in animals (Table 2), one might assume that its overactivation in PH is detrimental, and therefore should be pharmacologically suppressed. The effects of iNOS inhibitors in animal models were studied in in vitro and acute and chronic in vivo experiments. In some studies, the three non-selective NOS inhibitors L-NAME (N^ω^-nitro arginine methyl ester), L-NNA (N^ω^-nitro-L-arginine) and L-NMMA (N^ω^-monomethyl-L-arginine) were examined (given in italics in Table 3). In most of the studies shown in Table 3, however, selective iNOS inhibitors were examined, or the effects of selective and non-selective iNOS inhibitors were compared. Within the group of selective iNOS inhibitors, both competitive inhibitors, such as L-NIL (L-N^ω^-(1-iminoethyl)lysine), L-canavanine, and S-MIT (S-methylisothiourea), and non-competitive inhibitors, including 1400W, ONO-1714, and GW27415, were used [7].

With respect to in vitro experiments, it is not surprising that the direct influence of iNOS inhibitors on lungs or pulmonary vasculature was investigated, because all current state-of-the-art medications, approved for PAH treatment, are targeted towards pulmonary vasodilatation. Most experiments were performed on isolated tissues from animal models mimicking group 1 PH; the study by Morales-Cano et al. (2019; model mimicking group 2 PH) was the only exception [133]. Table 3 shows that non-selective NOS inhibitors had detrimental effects, including increased PAP, PVR, vascular tension, and exacerbated vasoconstrictor responses, independently of the experimental model [115,132,151,152,153,154]. This may be caused mainly by the inhibition of eNOS. However, in lungs from chronically hypoxic rats, as opposed to MCT-treated animals, no increase in PAP after L-NAME administration was observed [151].

**Table 3:** The efficacy of inducible nitric oxide synthase (iNOS) inhibitors in preclinical models resembling different clinical groups of pulmonary hypertension.

Disease Entity	Model(s)	Species	Selective and Non-Selective iNOS Inhibitor(s)	Acute/Chronic/ In Vitro	Preventive (P)/Curative (C) Treatment	Effects	References
**Models of disease entities within group 1 PH**							
PAH	MCT- and Hx-induced PH	rat	*L-NAME*	in vitro	-	isolated lungs: MCT: ↑ basal PAP; ↔ ampl. of HPV Hx: ↔ basal PAP; ↔ ampl. of HPV	[151]
						isolated PA (MCT and Hx): slight ↑ basal tension slight ↑ of Phe-induced constriction	[152]
	Hx-induced PH	mouse	*L-NAME*	in vitro	-	isolated PA: (-) Ach-induced relaxation ↔ PGF2α-induced contraction	[153]
		rat	L-NIL *L-NNA*	in vitro	-	isolated lungs: ↔ (L-NIL, *L-NNA*) basal PVR, arterial and venous resistance ↔ (L-NIL)/↑ (*L-NNA*) of arterial and (weak) venous constrictor responses to TXA_2_ analogue	[115]
PPHN	Hx-induced PH	pig	*L-NAME* AG	in vitro	-	isolated PA: ↓ (*L-NAME*)/↔ (AG) PA diameter	[154]
		rat ♀	*L-NNA*	in vitro	-	isolated lungs (from Hx neonates): ↑ PVR, ↑ reactivity to TXA2 analogue ↔ pulmonary venous tone	[132]
PAH	Sugen-Hx-induced PH	rat	L-canavanine	acute	-	8 weeks after Sugen-Hx: ↔ RVSP, ↔ LVSP, ↔ PVRI, ↔ SVRI	[155]
			*L-NAME*	acute	-	3, 5, and 8 weeks after Sugen-Hx: ↑ RVSP, ↑ LVSP, ↑ PVRI, ↑ SVRI, ↓ CI	
	Hx-induced PH	rat	S-MIT L-canavanine	acute	-	↔ PAP, ↔ SAP slight ↓ PAs (mainly muscular) diameter	[146]
			*L-NAME* *L-NMMA*	acute	-	↑ PAP, ↑ SAP ↓ PAs (muscular and elastic) diameter	
			ONO-1714	acute	-	↑ mPAP (slight and transient)	[145]
	Hx-induced PH	rat	L-NIL, *L-NAME*: (1) 3 days before + during a 1-week Hx; (2) 3 days before + during 1 week of a 3-week Hx; (3) during the final 10 days of a 3-week Hx	chronic	P (1,2); C (3)	L-NIL: ↔ SAP (1,2,3), ↓ PAP (1,2,3), ↓ exhaled NO (1,3), ↔ RV weight (1,3), ↓ RV weight (2), ↔ FI (1,3), ↓ FI (2) *L-NAME*: ↑ SAP (1,3), ↓ PAP (1,2) ↑ PAP (3), ↓ exhaled NO (1,3), ↓ RV weight (3)	[100]
		rat	ONO-1714 (10 days)	chronic	P	↔ mPAP, ↔ Hx-induced changes in vascular structure, ↔ FI	[145]
			*L-NAME* (4 weeks)	chronic	P	↓ PAP, ↑ SAP ↓ RV/BW, ↓ LV/BW	[110]
	MCT-induced PH	rat	AG (4 weeks)	chronic	P	↓ RVP restoration of Ach-induced relaxation (in PAs and systemic arteries)	[156]
PPHN	Hx-induced PH	pig ♀	L-NIL (10 days, 4 days after Hx onset)	chronic	C	**in fetal hearts:** anti-nitrative: ↓ 3-NT anti-oxidative: ↓ MDA anti-fibrotic: ↓ MMP-9, collagen other: ↓ cGMP levels	[142]
**Models of disease entities within group 2 PH**							
HFpEF	metabolic (obesity)-HF	rat	1400W	in vitro	-	isolated PA: modest ↑ Phe-induced vasoconstriction (only to its highest concentration)	[133]
	TAC	mouse	1400W (2 weeks)	chronic	C	↔ LV systolic pressure, ↔ HR LV hypertrophy and dysfunction: ↓ ventricular weight/BW ratio ↓ LV end-systolic diameter, ↑ LV ejection fraction, ↑ LV fractional shortening ↓ LV fibrosis pulmonary congestion: ↓ lung weight/BW ratio	[147]
	high-fat diet + L-NAME	mouse	L-NIL (3 days)	chronic	C	↔ HR, ↔ SBP, ↔ DBP, ↑ cardiac diastolic function (↓ E/A and E/E’ ratios), ↔ ejection fraction, ↑ running distance, ↔ lung edema (wet weight/dry weight ratio), ↔ heart weight/tibia length ratio oxidative status: ↓ MDA, ↑ GPX, ↓ NOX-4 ↑ pNRF2, ↔ SOD2, ↑ HO1 in hearts	[28]
	high-fat high-sucrose diet (HFHSD)	mouse	1400W (8 weeks)	chronic	C	↓ cardiovascular oxidative stress ↑ myocardial perfusion reserve ↓ arteriolar reactivity (-) HFHSD-induced ↓ in EF and changes in systolic and diastolic strain	[22]
**Models of disease entities within group 3 PH**							
COPD	cigarette smoke-induced lung injury	sheep ♀	MEG	acute	-	↓ PVRI, SVRI, ↑ CI, ↓ lung weight ↔ PAP, MAP	[157]
		mouse	L-NIL: (1) parallel to smoke exposure (8 months) (2) after 8 months of smoke exposure (3 months)	chronic	(1) P (2) C	↓ RVSP anti-hypertrophic: ↓ FI anti-emphysematic: ↓ mean linear intercept ↓ air space, ↑ septal wall thickness ↓ alveoli/vessels ratio	[119]
		guinea pig	L-NIL: (1) 7 days before smoke exposure (2) 60 days from the 29th day after smoke exposure	chronic	P	anti-emphysematic: ↓ mean linear intercept ↓ destructive index anti-oxidative: ↓ protein nitration and oxidation (lungs) anti-inflammatory: ↓ leukocyte infiltration, IL-1β, IL-8, TGF-β, IL-4 (BAL) ↓ total NOx (heart, liver, BAL)	[149]
					C	↔ mean linear intercept, ↔ destructive index	
	elastase-induced lung emphysema	mouse	L-NIL (12 weeks, 3 weeks after elastase instillation)	chronic	C	↓ RVSP, ↔ SAP, ↔ FI anti-nitrative (lungs): ↓ 3-NT ↓ iNOS anti-inflammatory: ↓ immune cells (CD45+), ↔ TNF-α, ↔ MMP-8, 9, 12 lung structure and function: ↓ pulmonary vascular muscularization	[141]
			1400W (20 days, 1 day before elastase instillation)	chronic	P	anti-nitrative: ↓ 3-NT (lungs) pro-oxidative: ↑ protein carbonyls other: ↔ mean chord length of alveoli ↔ HO1, MMPs, CCL-2, CXCL2, TNF-α, and IL-6 (lungs) ↔ inflammatory cell counts, CCL-2, MMP-2, MMP-9 protein (BAL) ↔ alveolar cell proliferation	[103]
COPD/IPF	SP-D deficiency-related emphysema	mouse	1400W (7 weeks from 3 weeks of age)	chronic	C	anti-oxidative: ↓ % of oxidants-producing macrophages anti-inflammatory (time-dependent): ↓ cellular infiltration, ↓ total BAL cell count, ↓ IFN-γ in BAL, ↓ macrophage recruitment anti-fibrotic: ↓ MMP-2, MMP-9	[150]
IPF	bleomycin (BLM)-induced lung injury	mouse	1400W (6 days before BLM instillation)	chronic	P	anti-nitrative: ↓ SNO-SP-D (BAL) anti-inflammatory: ↓ BAL chemotactic activity ↓ IL-1β, COX-2, CCL2 anti-fibrotic: ↓ Fizz1, TGF-β, Ym-1	[158]
			GW274150 (14 days, 1 day after BLM instillation)	chronic	P	anti-oxidative (lungs): ↓ lipid peroxidation anti-inflammatory (lungs): ↓ neutrophils infiltration anti-fibrotic (lungs): ↓ collagen formation and deposition ↓ TGF-β expression other: ↓ lung injury, ↓ edema formation, ↓ mortality rate; ↓ BW loss	[159]
		rat	AG (13 days, 1 day after BLM instillation)	chronic		anti-oxidative: ↓ MDA (pulmonary blood) * anti-nitrative: ↓ NOx (plasma) ‡ ↓ ONOO^-^ formation †,‡ anti-fibrotic: ↓ α-SMA and myofibroblast number ‡ ↓ type I ‡ and III † collagen lung deposition	[160] *, [161] †, [162] ‡
**Models of disease entities within group 4 PH**							
CTEPH	application of embolizing particles	chicken	AG	acute	-	↔ PAP, ↔ PVR, ↔ mortality	[163]
			*L-NAME*	acute	-	↑ PAP, ↑ PVR, ↑ mortality	
		dog ♀	AG	acute	-	↓ mPAP, ↓ PVRI	[164]
			S-MIT	acute	-	S-MIT: ↔ mPAP, ↔ PVRI (-) embolization-induced ↑ NOx, MDA, TBARS (plasma)	[165]
						S-MIT with sildenafil: ↓ mPAP, ↓ PVRI, but effect of sildenafil ↔	[164]
			*L-NAME*	acute	-	↑ mPAP, ↑ PVRI, ↑ mortality	[165]

Non-selective NOS inhibitors’ names are given in *italics*; experiments were performed on male animals, if not stated otherwise; ♀, female; 1400W, N-(3-(aminomethyl)benzyl)acetamidine; 3-NT, 3-nitrotyrosine; Ach, acetylcholine; AG, aminoguanidine; ampl., amplitude; BAL, bronchoalveolar lavage; BLM, bleomycin; BW, body weight; C, curative; CCL-2, small inducible cytokine A2; CD45+, cluster of differentiation 45 positive; cGMP, cyclic guanosine-3′,5′-monophosphate; CI, cardiac index; COPD, chronic obstructive pulmonary disease; COX-2, cyclooxygenase-2; CTEPH, chronic thromboembolic pulmonary hypertension; CXCL2, stroma-derived factor 1; EF, ejection fraction; FI, Fulton index; Fizz1, resistin-like molecule alpha 1; GPX, glutathione peroxidase; HFHSD, high-fat high-sucrose diet; HFpEF, heart failure with preserved ejection fraction; HO1, heme oxygenase 1; HPV, hypoxic pulmonary vasoconstriction; HR, heart rate; Hx, chronic hypoxia; IFN-γ, interferon gamma; IL-1β, -4, -6, -8, interleukin-1 beta, -4, -6, -8; iNOS, inducible nitric oxide synthase; IPF, idiopathic pulmonary fibrosis; *L-NAME*, Nω-nitro-L-arginine methyl ester; L-NIL, L-Nω-(1-iminoethyl)lysine; *L-NMMA*, Nω-monomethyl-L-arginine; *L-NNA*, Nω–nitro-L-arginine; LVSP, left ventricular systolic pressure; MCT, monocrotaline; MDA, malondialdehyde; MEG, mercaptoethylguanidine; MMP-2, -8, -9, -12, matrix metalloproteinase -2, -8, -9, -12; (m)PAP, (mean) pulmonary artery pressure; NO, nitric oxide; NOx, nitrite/nitrate; NOX-4, NADPH oxidase 4; ONOO-, peroxynitrite; P, preventive; PA(s), pulmonary artery(-ies); PAH, pulmonary arterial hypertension; PH, pulmonary hypertension; Phe, phenylephrine; pNRF2, phosphorylated nuclear factor erythroid 2-related factor 2; PPHN, persistent pulmonary hypertension of the newborn; PVR(I), pulmonary vascular resistance (index); RV(S)P, right ventricular (systolic) pressure; RV, right ventricle; SAP, systemic arterial pressure; S-MIT, S-methylisothiourea; SNO-SP-D, S-nitroso-surfactant protein-D; SOD2, superoxide dismutase 2; SP-D, surfactant protein-D; SVR(I), systemic vascular resistance (index); TAC, transverse aortic constriction; TBARS, thiobarbituric acid reactive substances; TGF-β, transforming growth factor beta; TNF-α, tumor necrosis factor alpha; TXA2, thromboxane A2; Ym-1, chitinase-like protein 3; α-SMA, alpha smooth muscle actin; ↓, decrease; ↑, increase; ↔ no change(s); (-), inhibition; * Chen et al. (2001); † Chen et al. (2003); ‡ Chen et al. (2017).

Selective iNOS inhibitors, like L-NIL and AG, neither intensified pulmonary vascular responsiveness to vasoconstrictors, nor did they change the pulmonary artery diameter in hypoxia-induced PH (Table 3) [115,154]. Admittedly, the selective iNOS blocker 1400W slightly increased the pulmonary artery contractile response to phenylephrine, but this occurred at its highest (10 μM) concentration only [133]. One might thus assume that the detrimental effects of non-selective NOS blockade might be driven by the lack of function of the constitutive isoforms, rather than the inducible one.

Table 3 also shows the effects of the acute in vivo administration of iNOS inhibitors. This scheme of drug application is however very rarely encountered in clinical practice in the context of PH, since mainly chronic pharmacotherapy is carried out [1,4]. The findings from studies in which systemic iNOS inhibitors were administered acutely are consistent with those from the in vitro studies discussed above (Table 3). Non-selective NOS blockade by L-NAME or L-NMMA increased both pulmonary and systemic arterial pressure and vascular resistance indexes in rats with chronic hypoxia- and Sugen-hypoxia-induced PH [145,146,155], i.e., animal models mimicking group 1 PH. By contrast, selective iNOS inhibitors (i.e., L-canavanine, S-MIT (S-methylisothiourea), ONO-1714) did not influence the basal values of these parameters, despite a minor and temporary increase in mPAP induced by the latter compound [145]. As is noteworthy, these changes were likely independent from the stage of PH progression, since very similar increases were observed 3, 5, and 8 weeks after the onset of Sugen-Hx. The mechanism behind the L-NAME-induced increase in RV systolic pressure is apparently active pulmonary vasoconstriction, since an increase in cardiac output was not observed [155].

In the context of models mimicking group 3 PH, the acute administration of the selective iNOS inhibitor MEG (mercaptoethylguanidine) alleviated lung edema, and decreased pulmonary and systemic vascular resistance with no effects on pulmonary and systemic arterial pressure, in an ovine model of COPD [157]. Among the models corresponding to group 4 PH [84], the potential importance of blocking iNOS function was assessed by injecting intravenous microparticles (Table 3) into chickens [163] or dogs [164,165]. Similarly to the group 1 PH models, acute non-selective NOS inhibition increased PAP, PVR, and overall mortality (Table 3). Unlike L-NAME, AG successfully attenuated pulmonary vascular obstruction-induced PH [164]. However, S-MIT failed to reduce PH, although it decreased oxidative stress. mPAP reduction was achieved after the combined application of S-MIT and the phosphodiesterase-5 inhibitor sildenafil, but resulted solely from the effect of the latter [165].

With respect to preclinical research, the chronic in vivo administration of compounds to animals represents the most accurate approach in the context of PH. The studies addressing the prolonged administration of NOS can be divided into two main groups, in which either (1) preventive treatment (which was initiated before or parallel to PH induction) or (2) a curative approach (which was started at least two to three days after the onset of PH) was chosen (Table 3). This distinction was introduced for the purpose of this review, as the authors of the original studies either used these terms inconsistently or did not use them at all.

Direct comparisons between (1) and (2) were conducted by Hampl et al. (2006) [100] in a hypoxia-induced PH model and by Seimetz et al. (2011) [119] and Gupta et al. (2016) [149] in a cigarette smoke-induced COPD model (Table 3). The non-selective NOS inhibitor L-NAME, as expected, increased systemic BP, in both the preventive and curative protocols, and PAP in the curative one [100]. By contrast, the selective iNOS inhibitor L-NIL effectively decreased PAP with no impact on systemic BP in both protocols [100]. Moreover, it diminished RV systolic pressure and had anti-hypertrophic and anti-emphysematous effects [119]; anti-oxidative and anti-inflammatory effects occurred only if L-NIL had been administered preventively [149]. The question arises as to why the curative administration of L-NIL for two to three months was effective in mice [119], but not in guinea pigs [149]. In addition to the species difference, it is likely that the route of administration and dose may be decisive, i.e., 600 mg/mL in drinking water (p.o.) vs. 1 mg per animal via inhalation, respectively. Nevertheless, the reductions in the exhaled NO quantity [100], tissue nitrite/nitrate concentrations [149], and iNOS overexpression [141,149] further confirm the pharmacological efficacy of this compound. Analogous findings showing a high anti-nitrative potency were found also in an elastase-induced emphysema model, although RV hypertrophy was not alleviated [141]. What is more, a three-day curative application of L-NIL already improved the cardiac function and overall physical condition of mice with left heart failure, although PH development was not confirmed in this study [28]. The anti-oxidative, anti-nitrative, and anti-fibrotic potential of L-NIL was also seen in a porcine model of persistent PH in the newborn [142].

To the best of our knowledge, 1400W, another selective iNOS inhibitor, was not investigated in preclinical models of groups 1, 4, and 5 PH (Table 3). However, in a group 2 PH model, Zhang et al. (2007) [147] and Guo et al. (2023b) [144], using TAC- and high-fat high-sucrose diet-induced LV pressure overload models in mice, respectively, found beneficial anti-hypertrophic and anti-fibrotic effects of the chronic curative administration of this compound. Unfortunately, in none of the latter studies was PH development confirmed. Further, 1400W, administered preventively in bleomycin-induced pulmonary fibrosis [158] or curatively in surfactant protein-D deficiency-related emphysema [150], showed anti-nitrative, anti-inflammatory, and anti-fibrotic effects. Preventive 1400W administration in an elastase-induced emphysema model led to a combination of effects, including beneficial effects (slight anti-nitrative potency), detrimental effects (intensified protein carbonylation), or no effects at all (expression of inflammatory and emphysema-related parameters) [103].

Another three selective iNOS inhibitors were studied beyond L-NIL and 1400W (Table 3). GW274150 was investigated in a preventive model of pulmonary fibrosis. Its two-week preventive administration, apart from its anti-inflammatory, anti-oxidative, and anti-fibrotic effects, halted loss of body weight, alleviated lung injury, and decreased mortality [159]. AG was investigated in two preventive protocols. Four-week administration to rats with MCT-induced PH decreased RV pressure and restored MCT-abolished vasodilator responses in both pulmonary and systemic vessels [156]. In a pulmonary fibrosis model, anti-oxidative, anti-nitrative, and anti-inflammatory effects were observed [160,161,162]. ONO-1714 appeared ineffective, but this could be due to the short duration of its administration (10 days only) [145].

**Figure 2:**
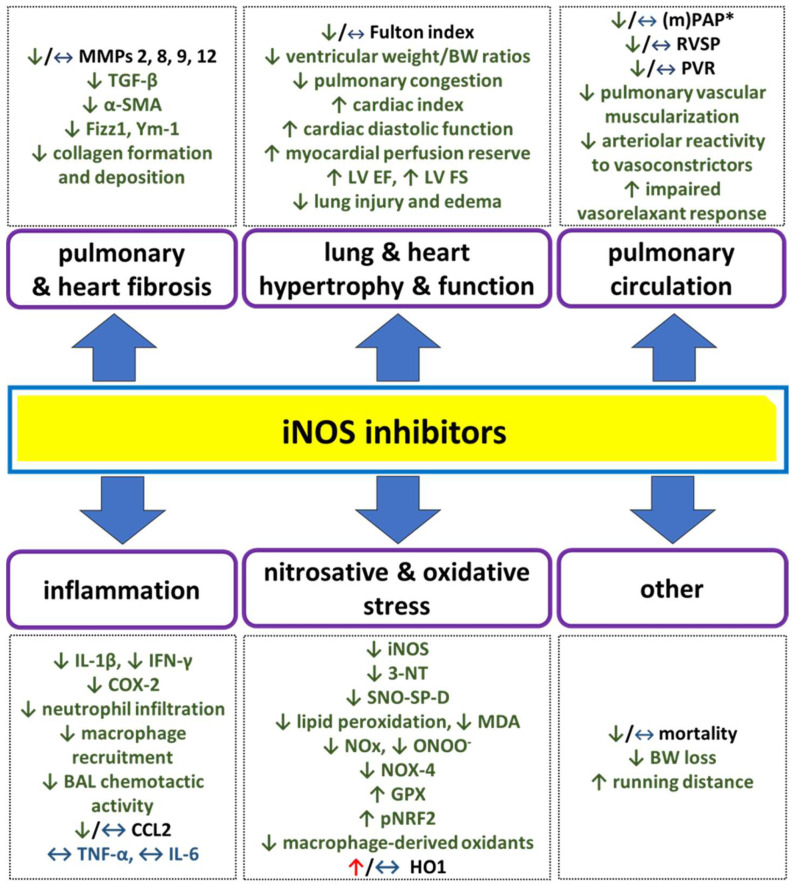
Effects of selective inhibitors of **inducible nitric oxide synthase** (**iNOS**) on key features of **pulmonary hypertension** (**PH**) and **PH-associated diseases** in the preclinical studies of Table 3. Changes in the discussed parameters which have a positive influence on PH progression are marked in green, those which might negatively affect the disease are shown in red, and those with a neutral effect in blue. Abbreviations: 3-NT, 3-nitrotyrosine; BAL, bronchoalveolar lavage; BW, body weight; CCL2, small inducible cytokine A2; COX-2, cyclooxygenase-2; EF, ejection fraction; Fizz1, resistin-like molecule alpha 1; FS, fractional shortening; GPX, glutathione peroxidase; HO1, heme oxygenase 1; IFN-γ, interferon gamma; IL-1β, -6, interleukin-1 beta, -6; iNOS, inducible nitric oxide synthase; LV, left ventricle; MDA, malondialdehyde; MMP-2, -8, -9, -12, matrix metalloproteinase-2, -8, -9, -12; (m)PAP, (mean) pulmonary artery pressure; NOx, nitrite/nitrate; NOX-4, NADPH oxidase 4; ONOO^−^, peroxynitrite; pNRF2, phosphorylated nuclear factor erythroid 2-related factor 2; PVR, pulmonary vascular resistance; RVSP, right ventricular systolic pressure; SNO-SP-D, S-nitroso-surfactant protein-D; TGF-β, transforming growth factor beta; TNF-α, tumor necrosis factor alpha; Ym-1, chitinase-like protein 3; α-SMA, alpha smooth muscle actin; ↑, increase/improvement of; ↓, decrease; ↔, no change; * ONO-1716 induced a slight and transient increase in mPAP after acute administration.

Figure 2 summarizes the main findings regarding in vivo and in vitro selective iNOS inhibition in the context of pulmonary hypertension (PH) and PH-associated diseases.

## 7. Is iNOS Gene Deletion Protective in PH? Insights from Knock-Out Studies

As discussed above, non-selective NOS inhibition resulted in a variety of detrimental consequences, including increases in systemic blood pressure and exaggerated vasoconstrictor responses. When considering the influence of NOS inhibition on PH progression, the question arises as to whether these effects were caused by the inhibition of the activity of different NOS isoenzymes, or rather by the unfavorable pharmacodynamic properties (off-target effects) of the inhibitors used. For this purpose, experiments on knock-out mice are very helpful. A variety of studies including global and conditional knock-out were taken into account (Table 4). Like non-selective pharmacological NOS inhibition, the deletion of all three NOS isoforms [166] increased RV systolic pressure [138], exacerbated cardiac and pulmonary artery hypertrophy and remodeling, intensified inflammation and fibrosis [138,167], and increased mortality [138]. This appears to be related to the lack of eNOS, since single eNOS knock-out did not appear preventive in the bleomycin-induced model of pulmonary fibrosis [166,167]. Moreover, it caused several detrimental effects in a hypoxia PH model [138]. The role of nNOS in the function of the cardiovascular system in the context of PH does not seem equally significant compared to the other isoforms, so we did not further consider its effects in this review [168]. However, one should keep in mind that in the knockout experiments, the lack of iNOS function may be compensated by other NOS isoforms [166,169].

The question of whether the selective pharmacological inhibition of iNOS (Table 3) generally reproduces the phenotype of iNOS knock-out mice (Table 4), despite the differences in the pharmacokinetic and pharmacodynamic properties of the agents, has been directly compared in seven studies (Table 3 and Table 4). Chronic GW274150 administration exactly mirrored the anti-inflammatory, anti-oxidative, anti-fibrotic, and other positive effects of iNOS knock-out mice in a bleomycin-induced lung injury model [159]. Moreover, the prolonged administration of L-NIL was as effective as genetic iNOS deficiency in alleviating oxidative stress, cardiac dysfunction, and pulmonary congestion in a model of HFpEF [28]. In a cigarette-smoke induced lung injury/COPD model, iNOS deficiency protected against the development of emphysema and PH; virtually the same results were obtained when wild-type (WT) mice were preventively or curatively treated with L-NIL [119]. The chronic administration of 1400W, like iNOS deficiency, led to similar anti-hypertrophic, anti-fibrotic, and anti-edematous effects in the TAC model [147], and improved perfusion reserve with reduced oxidative stress in the obesity-induced PH-LHD model [22].

On the other hand, although the pharmacological and genetic approaches inhibited oxidative parameters to a similar extent in a bleomycin-induced pulmonary fibrosis model, they differed in their effects on fibrosis; 1400W diminished but iNOS knock-out increased pro-fibrotic markers, suggesting that iNOS appears to be necessary for controlling the late-phase response to injury [158]. Finally, in an elastase-induced model of COPD, both pharmacological iNOS inhibition (by 1400W) and genetic iNOS deficiency appeared equally ineffective against a multitude of biochemical and histological markers, although both of them diminished protein nitration; both approaches also resembled each other inasmuch as they led to pro-oxidative effects [103].

As described above, iNOS expression occurs in different cell types that may be involved in PH pathogenesis and, for this reason, cell type-specific iNOS gene depletion is worth considering. Three studies have been published related to this topic (Table 4). (i) Alveolar epithelial type II cell-specific iNOS knock-out was ineffective in preventing an elastase-induced increase in RV systolic pressure, RV hypertrophy and dysfunction, and emphysema development [69]. (ii) By contrast, myeloid-cell-specific iNOS gene deletion effectively prevented the development of PH in the cigarette smoke-induced model of COPD, but not in the hypoxia-induced PH; moreover, protection against emphysema was not achieved [68]. (iii) It has already been mentioned that the development of PH and of emphysema was prevented in cigarette smoke-treated iNOS knock-out (iNOS^−/−^) mice. When the latter ones were transplanted with the bone matter of WT mice, the development of emphysema, as opposed to PH, was prevented. However, in wild-type mice transplanted with bone marrow from iNOS^–/–^ mice, protection against PH, but not against emphysema, took place [119].

Finally, the efficacy of iNOS knock-out across the different PH clinical groups and experimental models will be considered (Table 4; studies to groups 4 and 5 PH not available). No positive effects of either systemic or myeloid-cell specific iNOS gene deletion were observed in hypoxia-induced PH, a group 1 PH model (Table 4), despite the effectiveness of selective pharmacological iNOS inhibition (Table 3). However, in the models resembling group 2 PH (TAC and metabolic (obesity)-induced PH), a slight preventive effect of iNOS knock-out on, e.g., cardiac hypertrophy and dysfunction, oxidative stress markers and inflammation was observed (Table 4) [22,28,144,147]. However, in none of the latter studies was PH development confirmed by right heart catheterization or another relevant method. Moreover, RV function was not assessed. In group 3 PH models, the protection from cigarette smoke-induced emphysema [119] or PH development [68,119] in iNOS knock-out mice was very pronounced and substantially higher than in elastase-induced COPD models (Table 4). In the model of cigarette smoke-induced lung injury, changes in ferroptosis-related proteins were observed [148]. This finding is of interest, as the activation of ferroptosis may contribute to bronchoalveolar damage.

**Table 4:** Impact of global and conditional genetic deletion of inducible nitric oxide synthase (iNOS) * on pulmonary hypertension (PH): insights from preclinical murine models resembling different clinical PH groups **.

Disease Entity	Model	Effects				References
**Disease models within group 1 PH**						
PAH	Hx-induced PH	**iNOS^−/−^ vs. WT:**	**eNOS^−/−^ vs. WT:**		**triple n/i/eNOS^−/−^ vs. WT:**	[138]
		↔ RVSP; ↔ FI ↔ PA medial thickness ↔ survival rate	moderate ↑ RVSP; ↑ FI ↑ PA medial thickness ↓ survival rate		highest↑ RVSP; ↑ FI↑ PA medial thickness↓ survival rate	
		**myeloid-cell-specific iNOS^−/−^ vs. WT:** ↔ RVSP, ↔ FI, ↔ TAPSE, ↔ pulmonary vascular remodeling (small vessels)				[68]
**Disease models within group 2 PH**						
HFpEF	TAC	****iNOS**^**–/–**^**vs. WT:**** anti-oxidative: ↓ 4-HNE, ↓ 3-NT, ↓ PRMT1, ↓ DDAH1 in LV myocardium anti-hypertrophic: ↓ ventricular weight/BW ratio, ↓ myocyte cross-sectional area, ↓ myocyte diameter, ↓ MMP-2 and collagen-1 in LV myocardium ↓ cardiac dysfunction: ↑ LV ejection fraction, ↑ LV fractional shortening, ↑ LV diastolic wall thickness, ↑ LV diameter in end systole and diastole, ↓ cardiac ANP and BNP levels ↓ pulmonary congestion: ↓ lung weight/BW ratio other effects: ↔ mortality rate				[147]
		****iNOS**^**–/–**^**vs. WT:**** anti-inflammatory: ↓ cardiac IL-1β, IL-6 expression, CD68+ M1 macrophage count ↓ cardiac cytosolic mtDNA levels anti-fibrotic effects: ↓ fibrosis area ↓ cardiac remodeling and hypertrophy: ↓ myocyte cross-sectional area, ↓ heart weight/tibia length ratio ↓ cardiac dysfunction: ↓ cardiac ANP and BNP levels, ↑ LV ejection fraction, ↑ LV fractional shortening				[144]
	High-fat diet + L-NAME	****iNOS**^**–/–**^**vs. WT:**** (mostly) anti-oxidative: ↓ MDA, ↑ GPX, ↓ NOX-4, ↑ pNRF2, ↔ SOD2, ↑ HO1 in hearts ↓ cardiac dysfunction: ↑ cardiac diastolic function (↓ mitral E/E’ ratio) ↓ pulmonary congestion: ↓ lung edema (wet weight/dry weight ratio) other effects: ↑ running distance, ↔ heart weight/tibia length ratio, ↔ HR, ↔ ejection fraction, ↔ systolic BP, ↔ diastolic BP				[28]
	HFHSD	****iNOS**^**–/–**^**vs. WT:**** ↓ cardiovascular oxidative stress, ↑ cardiac stress perfusion ↑ vasodilatation to adenosine (in coronary arteries) ↓ HFHSD-induced changes in systolic and diastolic strain, ↔ cardiac rest perfusion, ↔ myocardial perfusion reserve, ↔ arteriolar reactivity				[22]
**Disease models within group 3 PH**						
COPD	cigarette smoke-induced lung injury	****myeloid-cell-specific iNOS**^**−/−**^**vs. WT:**** ↓ development of PH, but not emphysema ↓ RV hypertrophy, ↓ pulmonary vascular remodeling				[68]
		****iNOS**^**−/−**^**vs. WT:**** ferroptosis-related proteins: ↓ ACSL4, ↑ GPX4, xCT, FTL, FTH1				[148]
		****iNOS**^**−/−**^**vs. WT:**** protection against PH and emphysema				[119]
		**iNOS**^**−/−**^specific for bone marrow-derived cells *** vs. controls ****: ↓ PH: ↓ RVSP ↔ emphysema: ↔ number of alveoli		**iNOS**^**−/−**^except for bone marrow-derived cells ***** vs. controls ****: ↔ PH: ↔ RVSP ↓ emphysema: ↓ number of alveoli		
	elastase-induced lung emphysema	****iNOS**^**−/−**^**vs. WT:**** anti-nitrative: ↓ 3-NT positive cells pro-oxidative: ↑ protein carbonyls other: ↔ HO1, CCL2, CXCL2, TNF-α, and IL-6 (lungs) ↔ mean chord length of alveoli				[103]
		****AECII-specific iNOS**^**−/−**^**induced by doxycyclin vs. doxycyclin-naïve mice:**** ↔ RVSP, ↔ RV hypertrophy, ↔ FI, ↔ RVWT RV function: ↔ PAT/PET, ↔ TAPSE, ↔ pulmonary vascular muscularization emphysema development: ↔ lung compliance, ↔ mean linear intercept, ↔ lung airspace				[69]
IPF	bleomycin (BLM)-induced lung injury	****iNOS**^**−/−**^**vs. WT:**** anti-fibrotic: ↓ fibrosis score, ↓ TIMP-1, ↓ CCL-2, ↓ hydroxyproline content, ↓ α-SMA (lungs) other: ↔ lung compliance, ↓ mortality				[123,124]
		****iNOS**^**−/−**^**vs. WT:**** anti-nitrative: ↓ SNO-SP-D anti-inflammatory: ↓ chemotactic activity (BAL) ↓ IL-1β, COX-2, CCL-2 pro-fibrotic: ↑ Fizz1, TGF-β, Ym-1				[158]
		****iNOS**^**−/−**^**vs. WT:**** anti-oxidative: ↓ lipid peroxidation anti-inflammatory (lungs): ↓ neutrophil infiltration anti-fibrotic (lungs): ↓ collagen formation and deposition; ↓ TGF-β expression other: ↓ mortality rate, (-) loss of body weight, ↓ lung injury, ↓ edema formation				[159]
		****iNOS**^**−/−**^**vs. WT:**** anti-inflammatory: ↓ TNF-α, CCL-2, lymphocyte count, protein conc. (BAL), ↔ total inflammatory cells (BAL) anti-fibrotic (lungs): ↔ fibrotic area ↓ TGF-β1 ↓ collagen 1 other: ↔ BW, IL-1β, IL-6, IFN-γ, CTGF (BAL)	****eNOS**^**−/−**^**vs. WT:**** inflammatory parameters: ↔ TNF-α, CCL-2, IL-1β, IL-6, IFN-γ, CTGF, protein conc., lymphocyte count, total inflammatory cells (BAL) fibrotic parameters: ↔ lung fibrotic area ↔ TGF-β1 and collagen 1 in lungs other: ↔ BW		****triple n/i/eNOS**^**−/−**^**vs. WT:**** pro-inflammatory: ↑ TNF-α, CCL-2, IL-1β, IL-6, IFN-γ, lymphocyte count and protein conc. (BAL) ↑ total inflammatory cells (BAL) pro-fibrotic: ↑ lung fibrotic area, ↑ TGF-β1 ,↑ collagen 1, ↑ CTGF in lungs other: ↓ BW	[167]

* if not stated otherwise; ** if no additional information regarding the selectivity of gene deletion is included, the knock-out is global; *** iNOS^−/−^ specific for bone marrow-derived cells—WT mice transplanted with bone marrow from iNOS^−/−^ mice; **** controls—WT mice transplanted with bone marrow from WT mice; ***** iNOS^−/−^ except for bone marrow-derived cells—iNOS^−/−^ mice transplanted with bone marrow from WT mice; 3-NT, 3-nitrotyrosine; 4-HNE, 4-hydroxynonenal; ACSL4, long-chain fatty-acid-CoA ligase 4; AECII, alveolar epithelial type II cells; ANP, atrial natriuretic peptide; BAL, bronchoalveolar lavage; BLM, bleomycin; BNP, B type natriuretic peptide; BW, body weight; CCL-2, small inducible cytokine A2; CD68, cluster of differentiation 68; conc., concentration; COX-2, cyclooxygenase 2; CTGF, connective tissue growth factor; CXCL2, stroma-derived factor 1; DDAH1, dimethylamine dimethylaminohydrolase 1; eNOS, endothelial nitric oxide synthase; FI, Fulton index; Fizz1, resistin-like molecule alpha 1; FTH1, ferritin heavy chain; FTL, ferritin light chain; GPX(4), glutathione peroxidase (4); HFHSD, high-fat high-sucrose diet; HFpEF, heart failure with preserved ejection fraction; HO1, heme oxygenase 1; Hx, chronic hypoxia; IFN-γ, interferon gamma; IL-1β, -6, interleukin-1β, -6; iNOS, inducible nitric oxide synthase; IPF, idiopathic pulmonary fibrosis; L-NAME, Nω-nitro-L-arginine methyl ester; LV, left ventricle; MDA, malondialdehyde; MMP-2, matrix metalloproteinase 2; mtDNA, mitochondrial DNA; nNOS, neuronal nitric oxide synthase; NOX-4, NADPH oxidase 4; PA, pulmonary artery; PAH, pulmonary arterial hypertension; PAT, pulmonary acceleration time; PET, pulmonary ejection time; PH, pulmonary hypertension; pNRF2, phosphorylated nuclear factor erythroid 2-related factor 2; PRMT1, protein arginine methyltransferase 1; RV, right ventricle; RVSP, right ventricular systolic pressure; RVWT, right ventricular wall thickness; SNO-SP-D, S-nitroso-surfactant protein-D; SOD2, superoxide dismutase 2; TAC, transverse aortic constriction; TAPSE, tricuspid annular plane systolic excursion; TGF-β, transforming growth factor beta; TIMP-1, tissue inhibitor of metalloproteinase 1; TNF-α, tumor necrosis factor alpha; WT, wild type; xCT, cystine-glutamate antiporter; Ym-1, chitinase-like protein 3; α-SMA, alpha-smooth muscle actin; ↓, decrease; ↑, increase; ↔, no change(s).

## 8. iNOS Expression in Patients Affected by Diseases Associated with PH

iNOS is overexpressed during various cardiopulmonary disorders (see above), and as a mediator of inflammation it is also involved in the pathogenesis of PH. Amongst 36 studies on tissues obtained directly from humans (Table 5), 8 were related to PAH (clinical group 1), 18 to PH due to left heart disease (clinical group 2), and another 10 to chronic lung diseases and/or hypoxia (clinical group 3). Importantly, only parts of the subtypes/disease entities associated with PH were studied, i.e., congenital heart disease, persistent PH of the newborn, and PH due to congenital diaphragmatic hernia in group 1; heart failure and valvular heart disease in group 2; COPD and IPF in group 3 (Table 5). Furthermore, PH development was not confirmed in two studies from group 1 [170,171], 18 studies from group 2 [172,173,174,175,176,177,178,179,180,181,182,183,184,185,186,187], or in 8 studies from group 3 [68,123,124,188,189,190,191,192]. Ultimately, in some studies, the control groups could not be examined, the number of control individuals is not mentioned, or the studies are based on a very small number of patients only (as low as two to three per group; Table 5) [189]. These shortcomings render the discussion of a direct impact of iNOS overexpression on human PH very challenging.

**Table 5:** Organ/tissue/cell changes in inducible nitric oxide synthase (iNOS) expression in patients affected by pulmonary hypertension (PH) and/or diseases commonly associated with PH.

Disease Entity	Size of Sample	Number of Control Patients	Development of PH Confirmed? (+/-)	Changes in iNOS Expression (If Any) Versus Respective Control Group; Alteration of eNOS (If Studied)	References
Group 1 PH					
PAH associated with congenital heart disease (CHD)	18 (flow-associated PH) 6 (congestive vasculopathy) 10 (increased pulmonary blood flow but normal PAP)	4	+	↑ in PA ↔ eNOS	[193]
	26 (septal defects)	8	+	↑ in PA endothelium ↑ eNOS	[194]
	24 (VSD, including 10 surgically corrected)	-	+	↔ in lungs * ↓ eNOS	[195]
	7 (TOF); 8 (VSD)	-	TOF: -; VSD: +	detected in RA and RV myocardium	[196]
	19 (CHD)	10	-	↑ in LV myocardium **	[170]
rapid persistent PH of the newborn	2 neonates	3 neonates	+	↔ in lungs (PA endothelium, PA smooth muscle cells, macrophages, epithelium)	[189]
PH associated with congenital diaphragmatic hernia (CDH)	33 (10 ECMO-treated and 23 not treated by ECMO)	11	+	in small PA endothelium: ↔ treated by ECMO, ↓ not treated by ECMO ↔ eNOS	[197]
	13 (PH-CDH); 20 (lung hypoplasia due to other causes)	33	-	↔ in lung vasculature	[171]
**Group 2 PH**					
PH associated with left heart disease (PH-LHD)	43	15	+	↑ in monocytes	[198]
	20	15	+	↑ in PBMC	[199]
	15 (decompensated HF)	6	-	↑ in venous endothelium ↔ eNOS	[172]
	24 (DCM); 17 (IHD); 10 (VHD)	11	-	↑ in heart	[173]
	9 (HF—transplant group); 10 (LVAD); 11 (post-LVAD transplantation)	7	-	↑ in heart (HF-transplant and LVAD groups)	[174]
	28	4	-	↑ in heart ↑ in macrophages ↑ eNOS in cardiomyocytes and subendocardial areas	[175]
	18 (DCM); 7 (ischemic cardiopathy and severe ventricular dysfunction); 4 (AMI)	11	-	↑ in myocardium	[176]
	8 (DCM); 14 (IHD)	-	-	↑ in myocardium ↑ in endothelium, vascular smooth muscle cells	[177]
	14 (DCM); 9 (ICM); 7 (PCM)	5	-	↔ in myocardium ↑ eNOS	[178]
	24 (end-stage HF)	5	-	↑ in LV ↓ eNOS	[179]
	10 (HF due to CAD)	10	-	↑ in RA ↓ eNOS	[180]
	19	20	-	↑ in macrophages	[181]
	25 (acute congestive HF)	?	-	↑ in plasma	[182]
	10	?	-	↑ in plasma	[183]
	40	20	-	↑ in plasma	[184]
	42 (HFpEF) 38 (HFrEF)	-	-	↑ in serum (HFpEF) ↑ eNOS (HFrEF)	[185]
	23 (LVAD implantation) 36 (elective heart transplantation)	-	-	detected in heart and blood vessels	[186]
	7 (end-stage HF)	-	-	detected in LV myocardium	[187]
**Group 3 PH**					
PH associated with chronic obstructive pulmonary disease (COPD)	11 (severe COPD)	13 ***	-	↑ in lungs (alveolar wall, alveolar macrophages, bronchial wall, adventitia of PAs, smooth muscle cells)	[188]
	10	10	-	↑ in lungs	[148]
	10	10	-	↑ in pulmonary macrophages	[68]
	10	11	-	↑ in airway inflammatory cells	[190]
	13 (severe COPD); 14 (mild/moderate COPD)	13 smokers, 11 non-smokers	-	↑ in bronchial submucosa and bronchoalveolar lavage (smokers)	[191]
	7 (normal BMI); 7 (low BMI)	-	-	↑ in skeletal muscles (low BMI)	[192]
PH associated with idiopathic pulmonary fibrosis (IPF)	17	21	+	↑ in PAs ↓ eNOS	[200]
	17	10	-	↑ in lungs	[123]
	12	6	-	↑ in lungs (fibrotic scars, thickened septa, fibroblast foci)	[124]
	48	21	+	↑ in lungs (macrophages, neutrophiles, alveolar epithelium, PA endothelium, PA smooth muscle cells) ↓ eNOS	[201]

* in comparison to patients with ventricular septal defect (surgically corrected or not); ** high expression in 16 CHD cases, low expression in 3 CHD cases; *** control smokers; no healthy control subjects; AMI, acute myocardial infarction; BMI, body mass index; CAD, coronary artery disease; CDH, congenital diaphragmatic hernia; CHD, congenital heart disease; COPD, chronic obstructive pulmonary disease; DCM, dilated cardiomyopathy; ECMO, extracorporeal membrane oxygenation; eNOS, endothelial nitric oxide synthase; HF, heart failure; HFpEF, heart failure with preserved ejection fraction; HFrEF, heart failure with reduced ejection fraction; ICM, ischemic cardiomyopathy; IHD, ischemic heart disease; iNOS, inducible nitric oxide synthase; IPF, idiopathic pulmonary fibrosis; LHD, left heart disease; LVAD, left ventricular assist device; P(A)H, pulmonary (arterial) hypertension; PA, pulmonary artery; PAP, pulmonary artery pressure; PBMC, peripheral blood mononuclear cells; PCM, postmyocarditis cardiomyopathy; RA, right atrium; RV, right ventricle; TOF, tetralogy of Fallot; VSD, ventricular septal defect; VHD, valvular heart disease; ↑, increase; ↓, decrease; ↔, no change(s); +, yes; -, no(ne); ?, not stated.

Despite these limitations, three types of comparisons are possible on the basis of the available data, as follows: (1) iNOS expression across the PH-associated disease states, (2) iNOS expression patterns in organs primarily affected by PH, and (3) expression of other NOS isoforms, particularly of eNOS. These issues will be addressed separately for groups 1, 2 and 3.

With respect to PAH (group 1), iNOS was overexpressed in lungs, pulmonary arteries, and airways in most cases (Table 5). This overexpression localized most markedly to the pulmonary artery endothelium [194]. Moreover, enlarged iNOS activity was also found in plexiform lesions and the endothelium of muscular pulmonary arteries, and also in objects with increased pulmonary blood flow but no PH [193]. This might suggest the role of shear stress and cyclic strain in pulmonary vascular iNOS induction. Moreover, extracorporeal membrane oxygenation (ECMO) treatment was identified as a trigger of iNOS induction [197]. An increase in iNOS expression is also likely for the right atrium and ventricle of patients suffering from tetralogy of Fallot (TOF) and ventricular septal defect (VSD); however, it could not be quantified since control patients could not be examined in that study. Myocardial iNOS levels did not differ between patients with TOF and VSD, suggesting that hypoxemia (typical for TOF) does not play a role in this respect [196]. In studies on patients with VSD [195], congenital diaphragmatic hernia [171], and rapid persistent PH of the newborn [189], no changes in pulmonary iNOS expression were detected. In one study dedicated to patients with congenital diaphragmatic hernia (and not treated with ECMO), iNOS expression in the endothelium of small pulmonary arteries was even decreased [197].

Considering the correlations between inducible and constitutive NOS isoforms, PAH associated with congenital heart disease (CHD) showed elevated levels of both iNOS and eNOS in pulmonary vascular endothelial cells, but not in other cell types, i.e., pulmonary macrophages, airway epithelium, and alveolar lining cells [194]. This might suggest a potential compensatory mechanism aimed at restricting the increase in PAP (in long-standing PH, however, the increase in eNOS is reversed into a decrease because endothelial damage occurs; as discussed in Hoehn et al., 2009) [194]. In the other three studies, in which iNOS and eNOS were compared, three different scenarios were found: iNOS was overexpressed and eNOS was unaffected [193], iNOS unaffected and eNOS decreased [195], and iNOS decreased but eNOS unaffected (patients without ECMO) [197].

With respect to congestive heart failure, valvular diseases, and other left heart pathologies (e.g., cardiomyopathy) that might lead to group 2 PH, iNOS expression was increased in hearts, macrophages, and blood vessels (endothelium and smooth muscle cells), irrespective of PH diagnosis [172,173,174,175,176,177,178,179,180,181,182,183,184,185,186,187,198,199]. This increase was correlated with an increased iNOS activity [179]. Moreover, the intensity of iNOS overexpression had a significant relationship with the New York Heart Association (NYHA) class—higher iNOS amounts were detected in patients from classes III (associated with a marked limitation of patients’ physical activity) and IV (inability to engage in any physical activity without experiencing discomfort), rather than I (no limitation of physical activity) and II (slight limitation of physical activity) [198]. Moreover, increased levels of iNOS protein were also observed during the decompensatory phase of HF, and were generally linked to a larger LV volume, deteriorated LV function [172,202], and a higher expression of proinflammatory cytokines [183]. Moreover, in those patients, iNOS activity showed a strong linear relationship with plasma brain natriuretic peptide (BNP) levels [199]. Elevated BNP levels are often associated with PH. When the condition returned to its compensated state or a left ventricular assist device implantation procedure had been performed, iNOS expression significantly decreased [172,174]. A similar direction of changes in iNOS expression/activity was observed in patients with cardiomyopathy [173,176,177,178], valvular heart disease [173], coronary artery disease [180], and ischemic heart disease [177], and this suggests that iNOS is linked to heart failure (HF) itself (also in quantitative terms, NYHA class), rather than being connected to the underlying cause of the HF.

With respect to inducible and constitutive NOS isoforms, disease entities that might lead to PH-LHD showed different expressions of the two enzymes. In one study, elevated levels of both iNOS and eNOS were observed in the heart [175]. On the other hand, two studies reported decreases in eNOS with concomitant increases in iNOS expression in left ventricle [179] and right atrium [180], respectively. The other reported cases are no changes in iNOS, eNOS increased [178], and iNOS increased but eNOS unchanged [172]. Interestingly, in patients with heart failure with a preserved ejection fraction, higher iNOS concentrations were detected compared to patients with heart failure with reduced ejection fraction. However, eNOS concentrations tended to change in an opposite direction [185]. Genetic polymorphism of iNOS and eNOS genes might also play a role in the severity of HF [203]. It is noteworthy that, in nonfailing hearts, both iNOS and eNOS expression were minimal or undetectable [173,174,175].

With respect to chronic lung diseases leading to group 3 PH, iNOS expression was increased in patients with COPD and IPF, regardless of PH diagnosis. This overexpression was localized to airways, pulmonary vasculature, and inflammatory cells (Table 5). The rate of peroxynitrite-derived protein nitration in lung tissue was found to be directly proportional to iNOS expression. In COPD patients, the above relationship was associated with lower values of forced expiratory volume in one second and forced vital capacity [188]. It is noteworthy that patients with IPF exhibit a decreased (or absent) expression of eNOS in the pulmonary artery in two studies on patients suffering from IPF [200,201].

The question arises as to whether the expression patterns would also be similar in the other PH clinical groups (i.e., 4 and 5). In general, there are few studies on this, which involve relatively small patient populations (frequently without controls and without confirmation of the development of PH) and do not address the overall complexity of PH pathobiology; so it would be inappropriate to draw hasty, generalized conclusions. Further studies are needed to fully elucidate this particular problem.

## 9. Limitations and Perspectives

The following limitations should be considered. Although there is much evidence for iNOS overexpression in PH animal models (Table 2), the amount of studies regarding group 2 and 4 PH is relatively small. Similarly, studies involving the administration of iNOS inhibitors were most frequently carried out for group 1 and 3 PH (Table 3). Moreover, most experiments were performed on male rodents, and this does not accurately reflect clinical conditions, as female individuals are more predisposed to PAH development [204]. There is also a need for more studies on larger animals, e.g., as they allow for chronic instrumentation and repeated measurements in CTEPH models [205]. Finally, chronic curative treatment protocols mimic the actual pharmacotherapy schemes in PH humans most closely, yet studies of that type have been carried out rarely (regarding group 1, PAH) or not at all (regarding group 4 PH; Table 3). We were not able to find studies on human tissues (Table 5) from patients of clinical group 4. Moreover, in the case of diseases that might lead to group 2 PH (exhibiting the highest incidence rate), only two studies were found in which PH development was confirmed. With respect to group 1, only patients with PAH related to congenital heart disease were examined. Moreover, the number of individuals in the studies was often low, the lack of control groups in some of the studies made the interpretation of the results very difficult, and PH development was not confirmed in many studies. The latter limitations have much to do with the fact that ethical issues have to be strictly obeyed in experiments on humans. This also explains why the direct effect of iNOS inhibitors was not assessed. The side effects and toxicity of the discussed compounds should also be taken into account. The clinical development of 1400W was halted due to such concerns, while the development of ONO-1714 was discontinued because of inadequate selectivity between iNOS and eNOS [10].

This does not mean that inhibiting iNOS activity is per se detrimental, as many benefits were shown in preclinical studies, extending far beyond the PH discussed in this review. One potential reason for the lack of success could be the differences in disease pathomechanisms across species, making preclinical animal models insufficiently predictive. Additional challenges include an inadequate degree of in vivo iNOS inhibition, as well as the lack of tissue specificity or limited bioavailability [206,207]. As a result, novel iNOS modulators are being developed [207]. These agents are often based on the lead structures of already-known compounds and act upon iNOS in a similar manner [208]. Moreover, nanotechnological preparations offer the chance to overcome the limited bioavailability of highly lipophilic iNOS inhibitors [206]. Another approach is to introduce combined therapy. The β_2_-adrenoceptor antagonist ICI 118551 and the iNOS inhibitor 1400W, despite being ineffective if administered separately, reduced the lipopolysaccharide-induced mortality in an animal model of shock when given in combination [209]. Sometimes, iNOS-modulating properties are combined with the affinity to other targets in one molecule. Such an approach is called *polypharmacology* [210]. The dual-target-directed ligand (*S*)-MRI-1867 (a combined cannabinoid CB_1_ receptor and iNOS blocker) is considered a promising drug candidate for pulmonary fibrosis associated with Hermansky–Pudlak syndrome [211]. Continued efforts to refine and optimize such compounds could pave the way for more effective and safer therapies in the future.

## 10. Conclusions

**Figure 3:**
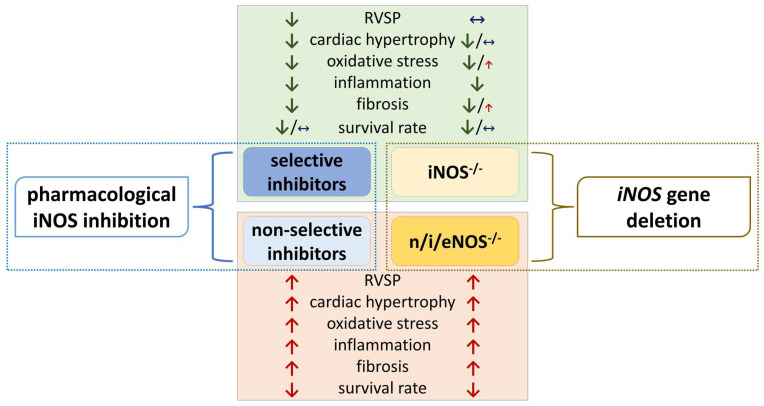
Effects of selective and non-selective inhibitors of **inducible nitric oxide synthase** (**iNOS**) and of genetic NOS depletion on key features of **pulmonary hypertension**. This diagram is based on the preclinical studies of Table 3 and Table 4. The smaller arrows depict changes that are divergent from the general trend. eNOS, endothelial nitric oxide synthase; nNOS, neuronal nitric oxide synthase; RVSP, right ventricular systolic pressure; ↑, increase; ↓, decrease; ↔, no change.

A detailed review of all publications regarding the role of iNOS in PH clearly shows that iNOS induction is associated with the pathogenesis of PH in its four main clinical groups and subtypes (according to the classification by Humbert et al., 2022; Table 1) [1]. In experimental animals and patients, iNOS is overexpressed in the organs primarily affected by PH (Table 2 and Table 5). The beneficial effects (see Figure 3) of suppressing iNOS activity by pharmacological inhibition (Table 3) or genetic depletion (Table 4) were disclosed across a variety of animal models of PH and PH-associated diseases, such as COPD or IPF. However, in some studies on iNOS knock-out animals, the direction of changes differed from the general trend (Figure 3). Only selective iNOS inhibitors are worth considering as a potential novel strategy for PH management. Non-selective NOS pharmacological blockade, like the simultaneous deletion of all three NOS isoforms, resulted in a variety of detrimental consequences, and even the aggravation of PH (Figure 3).

Although selective iNOS inhibitors are valuable pharmacological tools for studying the impacts of iNOS inhibition in various pathological conditions, none of the latter compounds have been marketed yet. However, two agents have been examined in phase II clinical trials on other indications, namely, cindunistat in symptomatic osteoarthritis of the knee [212], and GW274150 in rheumatoid arthritis [213] and migraine [214]. The possible implementation of selective iNOS inhibitors into the clinical management of PH appears to be rather far in the future. In the meantime, there is a need to decipher the interplay between the three NOS isoforms, iNOS-derived NO and inflammation, and between iNOS and other molecular signaling pathways in the context of all clinical groups of PH. Despite many promising results from preclinical studies, further attempts are needed to achieve the evaluation of iNOS-targeting drug candidates, overcoming the limitations discussed in Section 9, and the eventual optimization of current treatment strategies for pulmonary hypertension.

## Figures and Tables

**Table 1:** The clinical classification of the four main groups of pulmonary hypertension (PH), according to Humbert et al. (2022) [1]. Sub-groups, in which the role of inducible nitric oxide synthase (iNOS) was investigated, are marked in bold.

Clinical Groups	Classification
Group 1 pulmonary arterial hypertension (PAH)	**1. Idiopathic:** 1.1. Non-responders at vasoreactivity testing 1.2. Acute responders at vasoreactivity testing 2. Heritable 3. Associated with drugs and toxins **4. Associated with:** 4.1. Connective tissue disease 4.2. Human immunodeficiency virus (HIV) infection ** 4.3. Portal hypertension** ** 4.4. Congenital heart disease** 4.5. Schistosomiasis 5. PAH with features of venous/capillary involvement **6. Persistent PH of the newborn**
Group 2 PH associated with left heart disease	**1. Heart failure:** **1.1. With preserved ejection fraction** **1.2. With reduced or mildly reduced ejection fraction** **2. Valvular heart disease** **3. Congenital/acquired cardiovascular conditions leading to post-capillary PH**
Group 3 PH associated with lung diseases and/or hypoxia	1. Obstructive lung disease or emphysema * 2. Restrictive lung disease ** 3. Lung disease with mixed restrictive/obstructive pattern 4. Hypoventilation syndromes **5. Hypoxia without lung disease (e.g., high altitude)** 6. Developmental lung disorders
Group 4 PH associated with pulmonary artery obstructions	**1. Chronic thromboembolic PH** 2. Other pulmonary artery obstructions

Group 5 according to Humbert et al. [1] (PH with unclear and/or multifactorial mechanisms) does not appear in this table. *, e.g., chronic obstructive pulmonary disease (COPD); **, e.g., idiopathic pulmonary fibrosis (IPF).

## Abbreviations

The following abbreviations are used in this manuscript:

1400W	N-(3-(aminomethyl)benzyl)acetamidine
AG	aminoguanidine
BMPR2	bone morphogenetic protein receptor 2
cGMP	cyclic guanosine-3′,5′-monophosphate
CHD	congenital heart disease
COPD	chronic obstructive pulmonary disease
COX-2	cyclooxygenase-2
CTEPH	chronic thromboembolic pulmonary hypertension
ECMO	extracorporeal membrane oxygenation
EMA	European Medicines Agency
eNOS (NOS3)	endothelial nitric oxide synthase
FDA	Food and Drug Administration
FI	Fulton index
HF	heart failure
HFpEF	heart failure with preserved ejection fraction
HIF-1α	hypoxia-inducible factor 1 alpha
HO1	heme oxygenase 1
HPV	hypoxic pulmonary vasoconstriction
Hx	chronic hypoxia
IFN-γ	interferon gamma
IL-1β	interleukin-1 beta
iNANC	inducible non-adrenergic-non-cholinergic autonomic system
iNOS (NOS2)	inducible nitric oxide synthase
IPF	idiopathic pulmonary fibrosis
L-NAME	N^ω^-nitro-L-arginine methyl ester
L-NIL	L-N^ω^-(1-iminoethyl)lysine
L-NMMA	N^ω^-monomethyl-L-arginine
L-NNA	N^ω^–nitro-L-arginine
LPS	lipopolysaccharide
LV	left ventricle
MCT	monocrotaline
MEG	mercaptoethylguanidine
(m)PAP	(mean) pulmonary artery pressure
nNOS (NOS1)	neuronal nitric oxide synthase
NO	nitric oxide
NOS	nitric oxide synthase
NYHA	New York Heart Association
PA(s)	pulmonary artery(-ies)
P(A)H	pulmonary (arterial) hypertension
PH-LHD	pulmonary hypertension due to left heart disease
PVR(I)	pulmonary vascular resistance (index)
qPCR	quantitative polymerase chain reaction
RT-PCR	reverse transcription polymerase chain reaction
RV(S)P	right ventricular (systolic) pressure
RV	right ventricle
sGC	soluble guanylate cyclase
S-MIT	S-methylisothiourea
TAC	transverse aortic constriction
TGF-β	transforming growth factor beta
TNF-α	tumor necrosis factor alpha
TOF	tetralogy of Fallot
VEGFR	vascular endothelial growth factor receptor
VSD	ventricular septal defect
WT	wild type
